# Integrated Urinary and Tissue Proteomic Signatures Reveal Core and Progression Biomarkers in MRI-Visible and MRI-Non-Visible Prostate Cancer

**DOI:** 10.3390/life16030383

**Published:** 2026-02-27

**Authors:** Ana Blanca, Ana C. Morillo, Antonio Lopez-Beltran, Guillermo Lendinez Cano, Rafael A. Medina, Laura Chamorro Castillo, Daniel López Ruiz, Eduardo Chicano-Galvez, Juan Pablo Campos Hernández, Enrique Gómez Gómez

**Affiliations:** 1 Unit of Anatomic Pathology, Department of Morphological Sciences, Cordoba University Medical School, Av. Menéndez Pidal 7, 14004 Cordoba, Spain; em1lobea@uco.es; 2 Department of Urology, Maimonides Institute of Biomedical Research of Cordoba (IMIBIC), Reina Sofia University Hospital, University of Cordoba (UCO), Av. Menéndez Pidal, s/n, 14004 Cordoba, Spain; anac.morillo.sspa@juntadeandalucia.es (A.C.M.); laura.chamorro.castillo.sspa@juntadeandalucia.es (L.C.C.); juanp.campos.sspa@juntadeandalucia.es (J.P.C.H.); 3 Department of Urology, Virgen del Rocio University Hospital, Seville Biomedical Research Center (IBiS), Av. Manuel Siurot, s/n, 41013 Seville, Spain; guillermo.lendinez.sspa@juntadeandalucia.es (G.L.C.); rantonio.medina.sspa@juntadeandalucia.es (R.A.M.); 4 Department of Radiology, Maimonides Institute of Biomedical Research of Cordoba (IMIBIC), Reina Sofia University Hospital, University of Cordoba (UCO), 14004 Cordoba, Spain; danielj.lopez.sspa@juntadeandalucia.es; 5 IMIBIC Mass Spectrometry and Molecular Imaging Unit (IMSMI), Maimonides Institute of Biomedical Research of Cordoba (IMIBIC), Reina Sofia University Hospital, University of Cordoba (UCO), 14004 Cordoba, Spain; eduardo.chicano@imibic.org

**Keywords:** proteomic profiles, cancer visibility, prostate cancer, aggressiveness

## Abstract

**Background:** Prostate cancer (PCa) shows a marked biological heterogeneity that is closely associated with tumor aggressiveness. A substantial proportion of clinically significant tumors remain undetected by multiparametric magnetic resonance imaging (mpMRI). Elucidating the molecular basis of MRI visibility and identifying non-invasive biomarkers could improve the risk stratification and clinical management of patients. Accordingly, this study aimed to assess tissue and urine proteomic signatures associated with PCa aggressiveness and mpMRI visibility. **Methods:** In this exploratory study, we performed an integrated proteomic analysis of prostate tissue and preoperative urine samples from 24 patients stratified into four groups: benign prostatic hyperplasia (BPH), indolent PCa (Gleason 6), clinically significant PCa with MRI-visible lesions, and clinically significant PCa with MRI-non-visible lesions. Data-independent acquisition mass spectrometry (DIA workflows) was used to identify differentially expressed proteins associated with malignancy, tumor aggressiveness, and MRI visibility. **Results:** Pairwise proteomic analyses revealed significant molecular differences between BPH and all PCa groups, identifying 694 non-redundant proteins differentially expressed in tissue and 482 in preoperative urine, showing molecular features associated with both disease presence and progression. Comparative tissue and urine analyses identified 82 proteins, reflecting shared biological pathways in metabolism, cytoskeletal organization, immune processes, and extracellular matrix remodeling. Finally, a direct comparison of MRI-visible and MRI-non-visible clinically significant PCa identified a panel of differentially expressed proteins, including LCN2/NGAL, S100A9, and AOC1/DAO, that showed differential urinary abundance and prognostic relevance in the TCGA-PRAD cohort. **Conclusions:** Our results suggest that proteomic alterations in PCa are associated with disease progression and aggressiveness and capture biologically relevant differences between tissue and urinary proteomes. These differences are also observed between MRI-visible and MRI-non-visible clinically significant prostate cancers, supporting the potential of urinary proteomics as a non-invasive complement to imaging-based diagnostics.

## 1. Introduction

Prostate cancer (PCa) remains one of the most frequently diagnosed malignancies in men worldwide and is a leading cause of cancer-related morbidity and mortality [1]. Although there have been major advances in diagnostic imaging and clinical evaluation, the diagnostic workup for PCa remains incomplete. Prostate-specific antigen (PSA) testing is non-specific, leading to several undesirable consequences, including unnecessary biopsies, overdiagnosis, and overtreatment [2,3]. In recent years, multiparametric magnetic resonance imaging (mpMRI) has become increasingly part of the diagnostic workup before biopsy, enabling better lesion detection and risk stratification [4]. A recent three-year cohort study of 593 men found that nearly half (48%) had expert-interpreted negative mpMRI results and were able to avoid biopsy without increasing their risk. In this group, only 4% later developed clinically significant cancer, confirming that postponing biopsy after a negative mpMRI is safe when appropriate follow-up is in place [5].

However, although multiparametric MRI has improved the detection of clinically significant prostate cancer, it remains limited, as MRI visibility reflects an underlying biological phenotype rather than merely a technical constraint [4,6]. mpMRI detects fewer than half of all tumor foci and misses a notable share of clinically significant lesions, highlighting its limited per-lesion sensitivity. Smaller, low-grade, multifocal, non-index tumors with lower prostate-specific antigen density were more likely to be missed [7]. Some authors indicate that about one-third of biopsy-naïve men have a negative MRI, allowing many biopsies to be avoided and reducing overdiagnosis of ISUP grade 1 cancers by 18%. Nevertheless, systematic biopsy in MRI-negative men still detects ISUP grade ≥ 2 cancer in 8% of cases, meaning that roughly 12–13 men with a negative MRI would need to be biopsied to find one clinically significant cancer [8,9].

A further challenge in the treatment of PCa is the heterogeneity of clinically significant disease. Although the use of mpMRI has enhanced the detection of high-grade tumors, some clinically significant PCa remains invisible on MRI, potentially leading to underdiagnosis. Discovering molecular distinctions between MRI-visible and non-visible tumors may identify pathways that drive their imaging phenotypes and improve diagnosis. Therefore, our study focuses on characterizing these molecular signatures to improve the detection and management of both MRI-visible and MRI-non-visible csPCa, as well as distinguishing them from indolent PCa and benign hyperplasia.

Genomic and proteomic biomarkers for distinguishing benign prostatic hyperplasia (BPH) from PCa can be measured in tissue, blood, urine, and semen; however, the lack of standardized sample collection and analytical methods significantly limits their clinical reliability [10]. Validation of genomic and proteomic biomarkers, which are frequently combined with clinical data in multivariate panels, is required for their utilization in clinical practice. Proteomic approaches have been very useful for describing the molecular mechanisms underlying PCa development and progression, providing evidence of relevant metabolic and signaling changes [11].

Various tissue-based biomarkers have been investigated in PCa, including Bcl-2, Ki-67, EZH2, MCM proteins (particularly MCM7), and EIF3S (8q gain), providing insights into different aspects of tumor biology and prognostically helpful information for risk stratification [12]. Several tissue-based prognostic markers have been developed for clinical use and are now commercially available, including Prolaris, Decipher, and ProstaType. These tests combine gene expression signatures for cell cycle progression, metastatic potential, and tumor aggressiveness. This enhances risk stratification compared with conventional clinicopathological variables [13].

Specifically, tissue-based proteomic biomarkers have shown great potential for characterizing molecular alterations in PCa, revealing extensive dysregulation of protein complexes, with both low- and high-grade tumors showing distinct alterations in spliceosome-related, mitochondrial, anti-apoptotic, and integrin-associated complexes. These alterations represent sequential molecular changes within a tumor and provide insight into the structural and functional aberrations associated with PCa formation and progression [14]. However, tissue sampling is invasive because the prostate must be biopsied, and sampling error is an inherent limitation since PCa is often a multifocal and heterogeneous disease [15].

Conversely, because of its anatomical location and direct contact, urine is an easily accessible, non-invasive biofluid that can be used for early PCa detection and risk assessment. Examples include urine-based assays such as PCA3 and SelectMDx, which detect prostate-derived mRNA transcripts (e.g., PCA3, HOXC6, DLX1, KLK3) in post-DRE urine [12,16]. Furthermore, urinary proteomics profiles without DRE have already been validated in nearly 1000 patients at risk for PCa, demonstrating robust diagnostic performance [17].

In particular, urinary exosomal miRNAs, lncRNAs, circRNAs, and proteins provide a non-invasive means to distinguish metastatic from localized prostate cancer and reflect key mechanisms of tumor progression [12,18]. As a result, urine proteomics is a promising non-invasive approach for detection and risk stratification of PCa, complementary to mpMRI and clinical parameters, with validated CE-MS-based biomarker patterns demonstrating high diagnostic accuracy and good discrimination of clinically significant disease [17,19,20].

In this study, we performed an exploratory proteomic analysis of urine and prostate tissue samples across four clinically relevant groups: benign prostatic hyperplasia (BPH), Gleason 6 prostate cancer (PCa), clinically significant PCa (csPCa) with MRI-visible lesions, and csPCa with MRI-non-visible lesions. We aimed to identify differentially expressed proteins associated with malignancy (BPH vs. PCa), tumor aggressiveness (iPCa-Gleason 6 vs. csPCa), and MRI detectability in csPCa using high-resolution mass spectrometry. By comparing urinary and tissue proteomic landscapes, we examined whether urinary signatures reflect molecular changes in prostate tissue and thus whether they can be used to identify non-invasive biomarkers.

## 2. Materials and Methods

### 2.1. Study Design and Patient Cohort

This study was designed as an exploratory discovery analysis. We conducted a retrospective, analytical study including patients who underwent surgery for benign prostate hyperplasia (BPH) and prostate cancer (PCa) at Reina Sofía University Hospital (Córdoba, Spain) between 2018 and 2020. The study aimed to assess tissue and urine proteomic signatures associated with PCa aggressiveness and mpMRI visibility. The study was approved by the local institutional ethics committee (Act 4843/nº 317). It was performed according to the Declaration of Helsinki, and written informed consent was obtained from all participants before enrolment in the study.

A total of 24 patients were included and stratified into four groups (n = 6 per group) based on histopathological diagnosis and mpMRI findings: (I) benign prostatic hyperplasia (BPH), (II) non-significant PCa (Gleason score 6, ISUP grade group 1), (III) clinically significant PCa visible on mpMRI, and (IV) clinically significant PCa non-visible on mpMRI. Baseline renal function parameters, including serum creatinine and estimated glomerular filtration rate (eGFR), were included for all participants (Table 1).

### 2.2. Tissue and Urine Sample Collection

A total of 30 mL of urine was collected before surgery (radical prostatectomy for PCa patients and retropubic simple prostatectomy for BPH patients), immediately aliquoted, and stored at −80 °C until analysis. No digital rectal examination (DRE) was performed immediately before urine collection. Samples were obtained using standard procedures to limit pre-analytical variations and processed in a manner that did not interfere with the routine diagnostic workflow. All specimens were pseudo-anonymized before proteomic investigation. Paired tissue samples were obtained at surgery, processed by the Cordoba Biobank, and classified as MRI-visible or MRI-non-visible based on preoperative mpMRI, with representative tumor areas selected under pathologist supervision.

### 2.3. Multiparametric MRI Acquisition and Interpretation

Prostate mpMRI was performed on 1.5T or 3 T scanners using a 128-channel acquisition platform and a 30-channel dedicated abdominal coil. A standardized prostate mpMRI protocol was used, consisting of high-resolution anatomical, diffusion-weighted, and dynamic contrast-enhanced imaging sequences. The protocol comprised axial, sagittal, and coronal T2-weighted imaging, an isotropic T2 SPACE acquisition, DWI with several b-values, ADC mapping, and DCE imaging using gadobutrol (1 mmol/mL) as the contrast media. All mpMRI studies were evaluated by experienced genitourinary radiologists using PI-RADS version 2.1. (scores 1 to 5). Lesions with PI-RADS ≥ 3 were considered MRI-visible. Tumors were classified as MRI-non-visible when no suspicious lesion was identified on mpMRI (PI-RADS ≤ 2), despite histopathological confirmation of clinically significant disease. Lesion size thresholds were not used as an independent criterion for MRI visibility.

### 2.4. Urinary Proteomic Sample Preparation

Protein from urine and tissue samples was precipitated with trichloroacetic acid (TCA) using the acetone precipitation method. Protein pellets were resuspended in RapiGest SF reagent (Waters) and quantified by microfluorimetric assay. The same amount of protein from each sample was digested with trypsin using standard procedures.

### 2.5. NanoLC–MS/MS and DIA Proteomic Analysis

Peptide mixtures were subjected to nano-liquid chromatography tandem mass spectrometry (nanoLC-MS/MS) on an Ekspert nanoLC 400 system (Eksigent, Dublin, CA, USA) coupled with a C18 reversed-phase column (ThermoFisher Scientific, Waltham, MA, USA). The eluted peptides were analyzed on a TripleTOF 5600+ mass spectrometer (SCIEX, Framingham, MA, USA) in data-independent acquisition (DIA) mode at the Proteomics Unit of IMIBIC (Córdoba, Spain). This DIA-MS strategy enabled global quantification of urine proteins and was reproducible across all study groups (PRIDE repository, accession number PXD074635).

### 2.6. Protein Identification and Quantification

Raw mass spectrometry output was processed using established data-independent acquisition pipelines for peptide identification and protein inference. Protein intensities were normalized before statistical analysis. Redundant protein identifiers were merged to obtain non-redundant protein lists for downstream analyses.

### 2.7. Statistical and Differential Expression Analysis

The proteomic data were processed and quantified using Spectronaut Software (version 14, Biognosys, Schlieren, Switzerland), and statistical analyses were performed using MetaboAnalyst (version 6.0). To address multiple testing, false discovery rate (FDR) correction was applied to the global ANOVA analyses across the four study groups, and proteins with FDR < 0.05 were considered statistically significant. Post hoc Fisher’s least significant difference (LSD) test was used to detect differences among groups. In contrast, pairwise comparisons were performed as exploratory analyses using fold-change thresholds (≥2.0) and raw *p*-values (≤0.1) to maximize sensitivity for candidate biomarker discovery. Overlap analysis was used to determine common and distinct protein signatures within and across groups, as well as between tissue and urine matrices.

### 2.8. Comparative Tissue–Urine Proteomic Analyses

Significantly deregulated proteins from all tissue-based comparisons and urinary analyses were consolidated, and duplicate identifiers were removed to generate non-redundant protein sets. Proteins detected in both tissue and urine were identified to define a subset with potential translational relevance.

Functional annotation of shared proteins was performed using curated biological categories to classify them into metabolic processes, cytoskeletal organization, vesicle trafficking, protein synthesis, immune-related functions, and extracellular matrix components.

### 2.9. Survival Analysis Using the TCGA-PRAD Cohort

For complementary external evidence, gene expression data from the TCGA Prostate Adenocarcinoma (PRAD) dataset were used to assess broader molecular associations and prognostic relevance in PCa. The genes corresponding to candidate proteins identified between csPCa-MRI-visible and csPCa-MRI-non-visible tumors in tissue and urine were evaluated at the transcriptomic level using the publicly accessible UALCAN platform (https://ualcan.path.uab.edu/analysis.html, accessed on 28 January 2026). Patients were stratified by Gleason score and gene expression (high vs. low). Kaplan–Meier survival analyses were performed to assess the correlation of these genes with overall survival, and the log-rank test determined significance. A *p*-value < 0.05 was considered statistically significant.

## 3. Results

### 3.1. Tissue Proteome: Pairwise Comparisons

**Figure 1:**
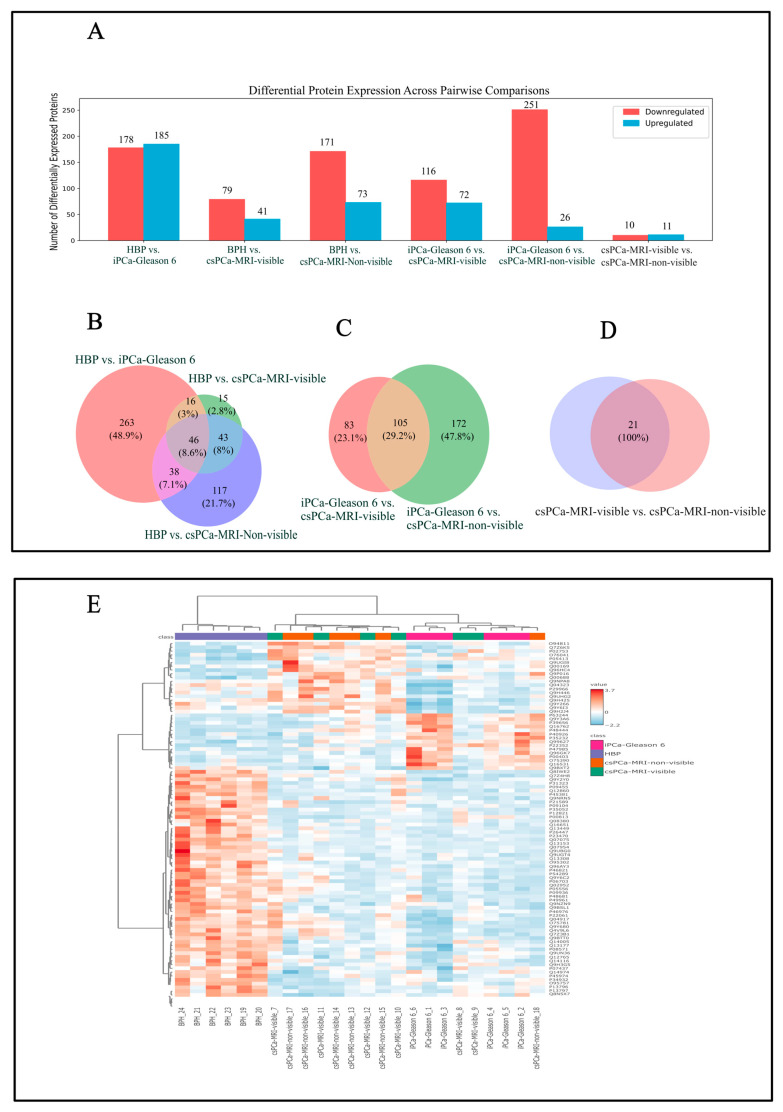
Differential tissue proteomic analysis across prostate cancer groups (**A**) Bar plot showing the number of significantly upregulated and downregulated proteins across pairwise tissue comparisons (fold change ≥ 2.0 and raw *p*-value ≤ 0.1). The comparison included benign prostate hyperplasia (BPH), iPCa Gleason 6, csPCa MRI-visible, and csPCa MRI-non-visible groups. (**B**–**D**) Venn diagrams illustrating the overlap of differentially expressed proteins among tissue comparisons, highlighting both shared and group-specific proteomic changes. (**E**) Hierarchical clustering heatmap of differentially expressed (ANOVA test; *p* < 0.05; FDR < 0.05) tissue proteins across all samples, demonstrating distinct proteomic patterns associated with disease state and MRI visibility. Color scale represents relative expression levels (red: upregulation; blue: downregulation).

Based on a threshold of fold change ≥ 2 and a raw *p*-value ≤ 0.1, differential expression analysis identified extensive protein deregulation across tissue samples. As shown in Figure 1, pairwise comparisons revealed extensive differential protein expression between benign (BPH) and malignant prostatic tissues. The comparative analysis between BPH and iPCa-Gleason 6 identified 363 differentially expressed proteins (178 downregulated and 185 upregulated). Additionally, comparisons between BPH and csPCa showed two distinct patterns: 120 differentially expressed proteins in MRI-visible csPCa and 244 in MRI-non-visible csPCa.

Within the malignant groups, comparisons between iPCa Gleason 6 and csPCa MRI-visible tumors identified 188 differentially expressed proteins. In contrast, comparisons with csPCa MRI-non-visible tumors revealed a larger shift, with 277 proteins differentially expressed.

Direct comparison between csPCa MRI-visible and csPCa MRI-non-visible tumors identified 21 differentially expressed proteins. After removing duplicate identifiers, all comparisons indicate that 694 unique, significant proteins were identified when combining all comparison groups (Figure 1A).

### 3.2. Tissue Proteome: Overlap and ANOVA

Comparative overlap analysis identified both shared and group-specific protein signatures across the study groups. A total of 46 proteins were commonly differentially expressed in comparisons between BPH and iPCa Gleason 6 and between BPH and csPCa, including both csPCa-MRI-visible and csPCa-MRI-non-visible groups (Figure 1B). In contrast, a larger set of 105 overlapping proteins was identified in comparisons between iPCa Gleason 6 and csPCa, irrespective of MRI visibility (Figure 1C). Notably, 21 proteins were identified exclusively in the direct comparison between csPCa MRI-visible and csPCa MRI-non-visible tumors (Figure 1D).

A global expression analysis based on analysis of variance (ANOVA) revealed a core set of 94 proteins that were significantly different across all study groups (BPH, iPCa Gleason 6, csPCa MRI-visible, and csPCa MRI-non-visible tumors; *p* < 0.05; FDR < 0.05). Post hoc Fisher’s least significant difference (LSD) testing revealed that approximately 80% of these proteins primarily discriminated benign tissue (BPH) from PCa, while a smaller subset (~20%) further differentiated tumor subtypes.

**Figure 2:**
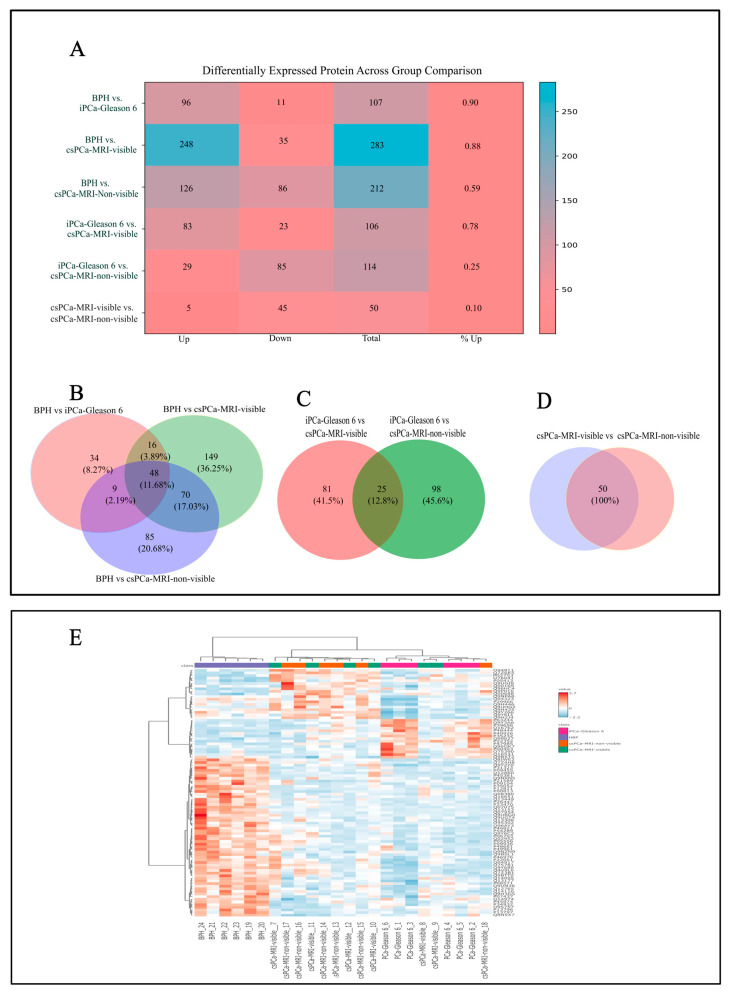
Analysis of differential urinary proteomic analysis across prostate cancer groups. (**A**) Heatmap summarizing the number of upregulated and downregulated urinary proteins across group comparisons (data from volcano plot; fold change ≥ 2.0 and raw *p*-value ≤ 0.1), including BPH, iPCa Gleason 6, csPCa MRI-visible, and csPCa MRI-non-visible tumors. (**B**–**D**) Venn diagrams depicting shared and unique differentially expressed urinary proteins across comparisons (**E**) Hierarchical clustering of urinary proteomic profiles (ANOVA test; *p* < 0.05; FDR < 0.05) showing clear separation of samples according to disease status.

As shown in Figure 2A, in urine samples from the same group of patients, the differential expression analysis using a volcano plot (fold change ≥ 2.0, raw *p*-value ≤ 0.1) revealed regulatory patterns across the study groups. The comparison between BPH and iPCa Gleason 6 identified 107 differentially expressed proteins, with a predominance of downregulated proteins. Comparisons between BPH and csPCa identified a larger number of upregulated proteins (248/283), especially in the csPCa MRI-visible group. When comparing iPCa Gleason 6 with csPCa, MRI visibility revealed opposite regulatory patterns: MRI-visible csPCa tumors showed the most upregulated proteins, whereas MRI-non-visible csPCa tumors showed the greatest contribution of downregulated proteins. Direct comparison between csPCa MRI-visible and csPCa MRI-non-visible tumors identified 50 differentially expressed proteins, the majority of which were downregulated in the csPCa MRI-non-visible group.

### 3.3. Urinary Proteome: Overlap and ANOVA

Across all pairwise comparisons involving BPH, 48 urinary proteins were consistently differentially expressed across PCa groups, including iPCa Gleason 6, csPCa MRI-visible, and csPCa MRI-non-visible. Pairwise analyses further identified 25 proteins that were consistently differentially expressed between iPCa Gleason 6 and both csPCa groups, regardless of MRI visibility.

After consolidation and removal of duplicate identifiers, a total of 482 unique significant proteins were identified across all urinary proteomic comparisons. These shared and group-specific urinary protein signatures are summarized in Figure 2B–D.

In parallel, urinary proteomic analysis using ANOVA identified 83 proteins that were significantly deregulated across the same four groups (Figure 2E).

Tissue–urine overlap: Comparative analysis of tissue and urine proteomes

**Figure 3:**
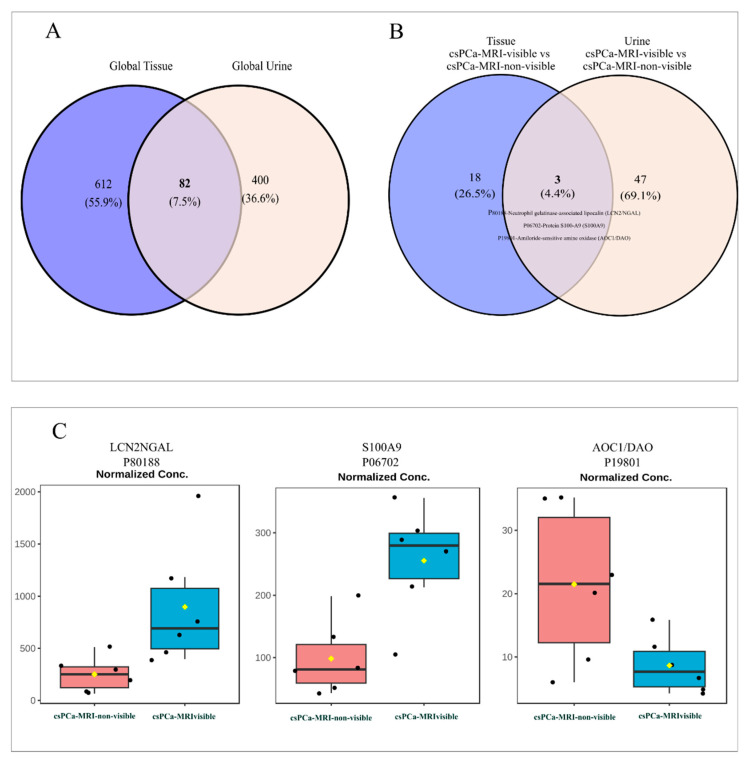
Comparison of tissue and urinary proteomes. (**A**) Venn diagram showing the overlap between the global tissue proteome and the urinary proteome. (**B**) Venn diagram comparing differentially expressed proteins between MRI-visible and MRI-non-visible csPCa in tissue and urine, identifying a subset of shared proteins (LCN2/NGAL, S100A9, and AOC1/DAO). (**C**) Box plots generated using MetaboAnalyst software 6.0 displaying normalized urinary expression levels of LCN2/NGAL (Raw *p*-value = 0.028), S100A9 (Raw *p*-value = 0.0004), and AOC1/DAO (Raw *p*-value = 0.036) in patients with MRI-visible and MRI-non-visible csPCa. Candidate proteins were selected based on differential expression analysis using volcano plot criteria (fold change ≥ 2.0; raw *p*-value ≤ 0.1). Black dots represent individual patient samples, and the yellow dot indicates the group means.

Comparative analyses of tissue and urine proteomes using 694 non-redundant significant tissue proteins and 482 urinary proteins identified 82 proteins (7.5%) shared between tissue and urine (Figure 3A) (Supplementary Table S1). These shared proteins represent a focused subset detectable in urine and reflective of tissue-level alterations. Functional annotation of the 82 shared proteins showed enrichment in pathways related to metabolic regulation, cytoskeletal organization, vesicle trafficking, and protein synthesis, as well as immune-related and extracellular matrix-associated processes. Proteins involved in antigen presentation (HLA-A-P19801), cell adhesion (ITGB1-P05556, CD44-P16070, MCAM-P43121), structural remodeling (LCP1-P13796, PLS3-P13797), extracellular matrix organization (LUM-P51884), and metabolic and redox regulation (SOD1-P00441, LDHA-P00338, PKM-P14618) were represented among this shared protein set (Table 2).

### 3.4. MRI-Visible vs. MRI-Non-Visible Overlap: Differential Urinary Protein Abundance

Venn diagram analysis comparing MRI-visible and MRI-non-visible csPCa revealed specific overlap between tissue and urine proteomes, with only three shared proteins (4.4%), compared with 18 tissue-specific (26.5%) and 47 urine-specific proteins (69.1%), underscoring a strong matrix-dependent proteomic signature (Figure 3B). The three proteins consistently detected in both tissues and urine were P80188-Neutrophil gelatinase-associated lipocalin (LCN2/NGAL), P06702-Protein S100-A9 (S100A9), and P19801-Amiloride-sensitive amine oxidase (AOC1/DAO). Their detection across both biological compartments suggests that a subset of molecular alterations related to csPCa is reflected in the urinary proteome. Urinary proteomic analysis revealed significant differences in the abundance of selected proteins between MRI-visible and MRI-non-visible prostate cancer cases (Figure 3C). Box plot comparisons showed higher urinary levels of LCN2 and S100A9 in patients with MRI-visible tumors than in those with non-visible disease. Additionally, AOC1/DAO showed higher expression in patients with csPCa-MRI-non-visible tumors than in those with MRI-visible tumors. These findings indicate that csPCa-MRI-visible may be associated with a distinct urinary proteomic profile, supporting the potential of these proteins as non-invasive markers of tumor visibility and biological aggressiveness.

To investigate matrix concordance in more detail, a paired correlation analysis between tissue and urine levels of LCN2, S100A9, and AOC1 was conducted using log2-transformed intensities (n = 12 paired samples). LCN2 (P80188) showed a moderate positive trend, and S100A9 (P06702) was not significantly associated. AOC1 (P19801) showed a significant negative Pearson correlation, but the Spearman correlation did not consistently support this. These results are shown in Supplementary Figure S1.

### 3.5. TCGA Validation

**Figure 4:**
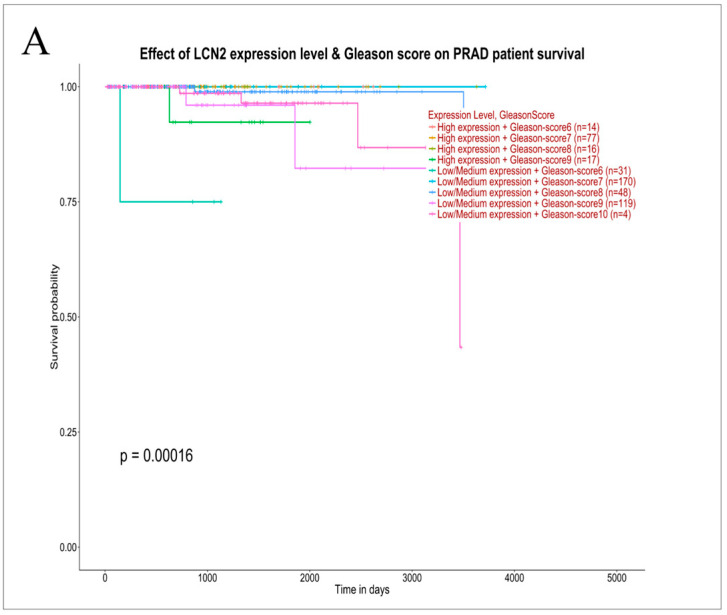

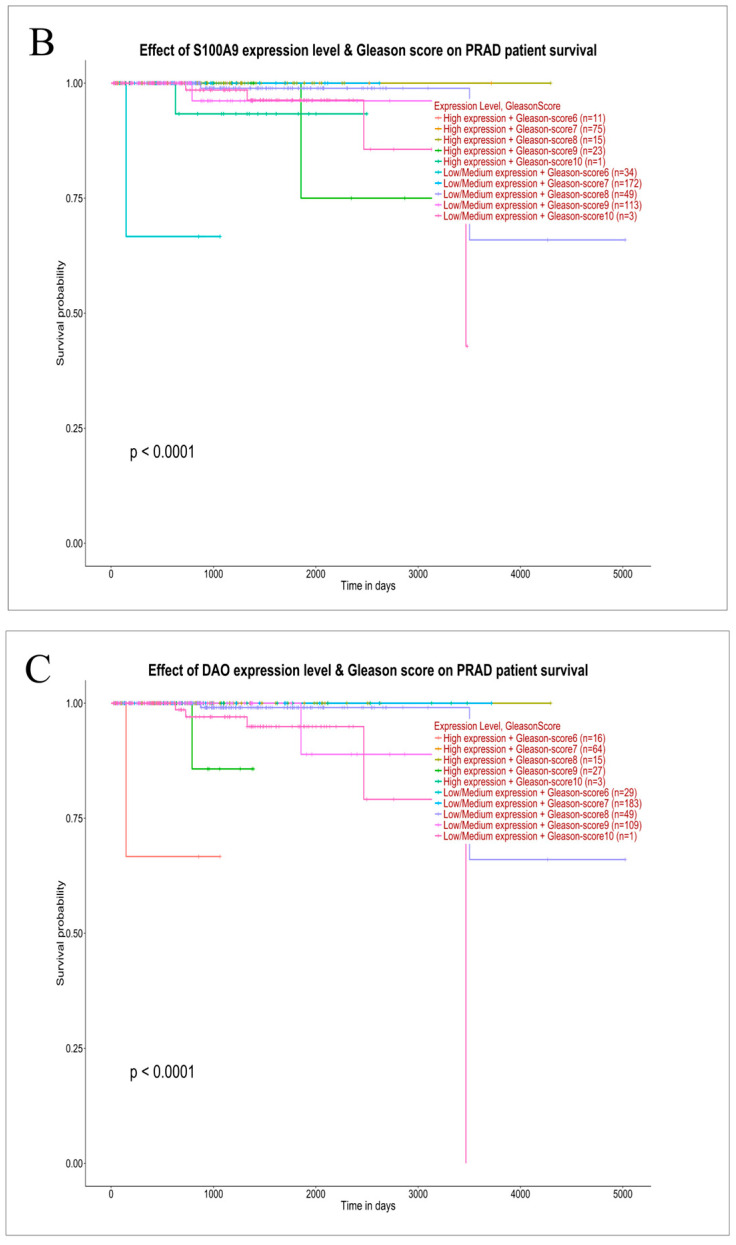
TCGA validation of the clinical relevance of proteins shared between tissue and urine. Kaplan–Meier survival analyses of prostate adenocarcinoma (PRAD) patients stratified by expression levels (low, medium, high) of LCN2 (**A**), S100A9 (**B**), and AOC1/DAO (**C**), in combination with Gleason score categories.

To further validate the clinical relevance of proteins shared between tissues and urine, the prognostic significance of LCN2/NGAL, S100A9, and AOC1/DAO was evaluated in the TCGA Prostate Adenocarcinoma (PRAD) cohort. Survival analyses were performed by stratifying patients by gene expression level (high vs. low) and Gleason score. Kaplan–Meier survival analysis revealed that patients with low or medium LCN2 expression, particularly those with higher Gleason scores, had reduced survival compared with those with high LCN2 expression (*p* = 0.00016; Figure 4A). Patients with low or medium S100A9 expression, especially at higher Gleason scores, had worse survival than those with high S100A9 expression (*p* < 0.0001; Figure 4B). Finally, patients with low or medium DAO expression tended to have reduced survival compared with those with high DAO expression, particularly in higher Gleason score categories (*p* < 0.0001; Figure 4C).

## 4. Discussion

In this study, we combined proteomic profiling of prostate tissue and urine across four clinically relevant groups: (I) benign prostatic hyperplasia (BPH), (II) indolent prostate cancer (iPCa; Gleason 6), (III) clinically significant prostate cancer with MRI-visible lesions (csPCa MRI-visible), and (IV) clinically significant prostate cancer with MRI-non-visible lesions (csPCa MRI-non-visible). This comparative proteomic approach revealed both shared and group-specific molecular signatures associated with malignancy, disease progression, and MRI detectability. These findings are in line with previous studies showing that proteomic alterations are associated with localized PCa and that distinct patterns of dysregulation differ between low- and high-grade tumors, thereby providing insight into the molecular changes underlying disease progression [11,14]. These data support a layered model of PCa biology, characterized by early, common alterations, gradual progression-related proteomic remodeling, and a distinct proteomic signature associated with MRI visibility, consistent with prior evidence indicating that mpMRI conspicuity reflects intrinsic tumor biology rather than technical factors alone [17,19].

Analysis of prostate tissue proteomes revealed extensive molecular differences between benign and malignant tissues, with many differentially expressed proteins already detectable in comparisons between BPH and iPCa Gleason 6 (363 differentially expressed proteins). These early alterations were shared mainly across PCa groups, suggesting that fundamental molecular remodeling occurs at early stages of malignant transformation [14,21]. In contrast, comparisons involving clinically significant tumors revealed additional proteomic changes, reflecting disease progression and increased biological complexity [15].

Significantly, although both csPCa MRI-visible and csPCa MRI-non-visible tumors shared a common progression-related signature, a limited subset of proteins differentiated the two groups (21 differentially expressed proteins). This finding highlights the biological heterogeneity of clinically significant PCa. It supports the concept that MRI visibility is not merely a technical phenomenon but reflects underlying molecular differences within the tumor and its microenvironment [15,22].

Urinary proteomic profiling revealed deregulation patterns that closely paralleled those observed in tissue samples, consistent with urine being collected from the same patients before surgical intervention. Consequently, urinary proteomics has gained increasing attention as a promising strategy for biomarker discovery, early detection, active surveillance, and risk stratification, with the potential to complement mpMRI and other established clinical tools. In this context, a proteomics-based 19-biomarker model (19-BM), developed using capillary electrophoresis–mass spectrometry (CE-MS), has already been validated in nearly 1000 patients at risk for PCa, demonstrating robust diagnostic performance [17]. Additionally, advances in mass spectrometry and bioinformatic analysis have increased the sensitivity and specificity of urinary proteomic profiling, enabling the detection of candidate biomarkers that discriminate csPCa from indolent disease and BPH [19,20]. These findings underscore the growing need for non-invasive biomarkers [23]. In this context, liquid biopsy, an approach that analyzes tumor-derived components in body fluids, has emerged as a promising diagnostic strategy for various cancers, including PCa [20]. A core set of urinary proteins consistently distinguished PCa from BPH, independent of Gleason grade or MRI visibility, indicating that urine captures stable molecular features associated with malignant transformation, as reported in previous urinary proteomic and extracellular vesicle-based studies [18].

Beyond this core signature, additional urinary protein subsets discriminated indolently from clinically significant disease and further distinguished MRI-visible from MRI-non-visible tumors (50 proteins were differentially expressed). These findings demonstrate that urinary proteomics not only reflects the presence of PCa but also captures disease heterogeneity and progression-related molecular changes [24,25].

The identification of a robust urinary signature distinguishing PCa from BPH underscores the potential of urine as a non-invasive source of diagnostic biomarkers [17,19]. Proteins shared across all PCa groups likely represent fundamental biological changes associated with tumor initiation [11,26]. In parallel, proteins shared between iPCa Gleason 6 and clinically significant disease suggest that molecular features related to aggressiveness emerge early during tumor evolution [14,27].

In contrast, the direct comparison of csPCa MRI-visible and MRI-non-visible tumors revealed distinct urinary proteomic profiles, indicating that MRI visibility is associated with specific molecular programs [22,28]. The predominance of downregulated proteins in MRI-non-visible tumors suggests a less active or differently organized proteomic landscape compared with MRI-visible disease [7,15].

The observation that MRI-visible tumors exhibit distinct proteomic features is consistent with prior tissue-based and multi-omic studies, demonstrating that mpMRI visibility reflects intrinsic tumor biology rather than tumor size alone [7,22,28]. Previous work by Houlahan et al. and others has shown that MRI-visible lesions harbor large-magnitude molecular differences, supporting a biological basis for radiological heterogeneity [28].

The identification of 82 proteins shared between prostate tissue and urine highlights a focused set of molecular alterations that are consistently detectable across both biological compartments. The functional enrichment of these proteins in pathways related to metabolic regulation, cytoskeletal organization, vesicle trafficking, protein synthesis, immune processes, and extracellular matrix remodeling suggests that key aspects of tumor biology are reflected in the urinary proteome [23]. Proteins involved in antigen presentation (HLA-A) [29,30] and cell adhesion (ITGB1, CD44, MCAM) indicate modified interactions between tumor and microenvironment [31,32], while structural and cytoskeletal components (LCP1, PLS3) support ongoing tissue remodeling [33,34]. The presence of extracellular matrix-associated proteins such as lumican (LUM), together with metabolic and redox-related enzymes (SOD1, LDHA, PKM), further indicates that metabolic reprogramming and oxidative stress are shared features of PCa biology detectable in urine [35].

Based on our results, the identified functional categories suggest that urinary proteomics captures core biological processes associated with tumor aggressiveness. Previous studies have shown that metabolic and redox-related proteins are associated with metabolic reprogramming [11,14,21]. In contrast, cytoskeletal and extracellular matrix-associated proteins may reflect increased cellular density, tissue remodeling, and tumor-stroma interactions, hallmarks of more aggressive PCa [14,21]. Collectively, these findings support the translational relevance of urinary proteomics as a non-invasive readout of tissue-level molecular alterations [23,36]. The overlap between tissue and urinary proteomes identified a focused subset of proteins detectable in both biological compartments, reinforcing the translational relevance of urinary proteomics.

The comparative analyses of MRI-visible and MRI-non-visible csPCa between tissue and urine revealed distinct molecular signatures associated with tumor visibility. This suggests that MRI-visible csPCa is characterized by a more pronounced inflammatory or immune-reactive microenvironment. Such biological features may contribute to MRI conspicuity by promoting tissue remodeling, increased cellularity, stromal reaction, or altered vascular permeability, all of which are known to influence MRI signal characteristics. These findings suggest that the group of patients with csPCa-MRI-visible tumors reflects intrinsic tumor biology rather than technical limitations, indicating that MRI-visible tumors may be characterized by a more inflammatory and immune-reactive microenvironment and by other mechanisms that contribute to mpMRI enhancement [22,28]. On the other hand, in csPCa-MRI-non-visible cases, these results could represent a distinct biological phenotype with diminished inflammatory infiltration or other, as yet unknown, metabolic adaptations that develop despite reduced radiological visibility and have clinical implications [7,22]. The detection of shared proteins such as LCN2, S100A9, and AOC1/DAO indicates that key tumor-associated biological processes in csPCa are reflected in the urinary proteome. These proteins are involved in inflammation and immune regulation, highlighting the potential of urine-based liquid biopsy to capture relevant aspects of tumor biology non-invasively. [19]. Among the subset of proteins consistently detected, LCN2 and S100A9 showed higher urinary abundance in patients with MRI-visible tumors than in those with MRI-non-visible disease. Both proteins are associated with inflammatory signaling and innate immune activation. In prostate tissue, NGAL expression has been reported by in situ hybridization [37]. Higher NGAL expression has been observed in acute and chronic inflammatory states and in various cancer types, including PCas [38]. In contrast, lower NGAL mRNA levels have been reported in metastatic tissues compared with primary tumors [39]. NGAL is also a recognized biomarker of renal tubular stress and AKI [37]. Thus, renal function may be a confounder in the analysis of urinary NGAL, specifically in an aged cohort. Baseline renal function (serum creatinine and eGFR) in our cohort was not suggestive of significant renal impairment. It was similar across groups, reducing the possibility that NGAL enhancement was mainly kidney-derived. Additionally, S100A9 is frequently overexpressed in PCa, among other cancers, suggesting a correlation with the mechanistic intricacies of carcinogenesis and the attenuation of cellular differentiation. Overexpression of S100A9 is associated with poor prognosis and increased tumor aggressiveness [40] and has also been reported in aggressive PCa and is associated with tumor-promoting inflammation [41]. In contrast, AOC1/DAO showed higher urinary levels in MRI-non-visible csPCa, suggesting a distinct biological phenotype in tumors that escape radiological detection. Recent studies indicate that AOC1 is downregulated in PCa, and these reduced levels are positively correlated with the tumor size, lymph node metastasis, and Gleason score, supporting a potential tumor-suppressive role. Additionally, AOC1 expression in PCa is positively regulated by the transcription factor SOX15, and both may be promising targets for PCa treatment [40]. In this context, the relative enrichment of DAO in MRI-non-visible tumors may reflect a biologically distinct subset characterized by preserved or alternative metabolic regulation rather than overt aggressive expansion. Considering the role of DAO in amine metabolism and the regulation of inflammatory mediators such as histamine, its relative enrichment in csPCa-MRI-non-visible may reflect a microenvironment with reduced inflammatory infiltration or alternative metabolic adaptations that do not generate strong imaging contrast and cellular density [42]. Therefore, increased DAO levels in csPCa MRI-non-visible tumors may reflect a microenvironment with reduced inflammatory infiltration, altered immune reactivity, or alternative metabolic-redox adaptations that do not produce the tissue density and vascular changes typically associated with strong mpMRI conspicuity. Although causal mechanisms cannot be established in this exploratory study, these findings support the hypothesis that MRI-non-visible tumors represent a metabolically and immunologically distinct phenotype.

Collectively, LCN2/NGAL, S100A9, and AOC1/DAO seem to reflect complementary inflammatory and metabolic aspects of csPCa; however, LCN2/NGAL is also affected by renal function, and kidney parameters were not significantly altered in our cohort. The combined analysis of LCN2/NGAL with S100A9 and AOC1/DAO might improve biological specificity for broader clinical applications.

At the clinical levels, urinary proteins associated with MRI visibility could serve two complementary roles. First, they may improve pre-imaging risk stratification by identifying patients who are more likely to harbor MRI-visible, clinically significant disease. Second, they could help detect clinically significant tumors that remain MRI-invisible, reducing false reassurance after a negative mpMRI. Importantly, this study was designed as an exploratory analysis, and the identified protein signatures should be interpreted as hypothesis-generating rather than definitive biomarkers. These findings align with emerging evidence supporting the integration of urinary biomarkers with imaging to optimize PCa detection and management, and to better understand the biological aggressiveness of visible and non-visible tumors. The distinct expression patterns of S100A9, LCN2/NGAL, and AOC1/DAO might reflect complementary biological processes and conditions, including tumor aggressiveness, imaging visibility, and possible contributions from renal sources. However, independent validation studies using a large cohort will be required before considering clinical implementation.

Gene expression data from the TCGA-PRAD cohort were analyzed to explore the clinical relevance of the selected candidates LCN2, S100A9, and AOC1/DAO. Notably, TCGA-based analyses do not constitute a direct validation of urinary protein abundance but rather provide complementary evidence of the biological and prognostic relevance of the three chosen candidates.

Nevertheless, given the exploratory nature of this study, further validation in larger independent cohorts is warranted, including an assessment of urinary markers alongside clinical parameters and validation of predictive models for MRI visibility and disease aggressiveness.

### Limitations

Certain limitations should be noted, including the small sample size (n = 24; six patients per group), which represents the main limitation of this study and restricts statistical power and generalizability of the findings, as well as the exploratory, discovery-driven nature of the study. Urinary proteomics can also be affected by non-tumoral factors, such as hematuria, inflammation, and sample processing, which may be responsible for some of the observed modifications. The functional annotation of urinary proteins should be interpreted with caution and further validated by orthogonal approaches, e.g., targeted mass spectrometry or immunohistochemistry. Finally, the study does not demonstrate causality between individual proteins and MRI conspicuity, and validation in an independent cohort is warranted.

## 5. Conclusions

This integrated tissue and urinary proteomic analysis showed that PCa progression and MRI visibility are associated with distinct molecular signatures. A core urinary proteomic signature distinguishes PCa from benign disease, while additional protein subsets capture disease aggressiveness and MRI detectability. These findings support the biological basis of mpMRI visibility and highlight the potential of urinary proteomics as a non-invasive complement to imaging for improved detection and stratification of csPCa. Finally, although our results provide a promising framework for non-invasive biomarker development, they should be interpreted as hypothesis-generating and require further validation in independent prospective studies before clinical application.

## Figures and Tables

**Table 1:** Baseline clinicopathological characteristics of the study cohort (N = 24).

Variable			Value	
Age, years			62.09 ± 6.07 (median: 63)	
Total, PSA, ng/mL			6.21 ± 3.52 (median: 4.9)	
Free PSA, %			13.88 ± 4.76 (median: 14)	
Prostate volume, cc			61.33 ± 40.32 (median: 50)	
**Benign prostatic hyperplasia (BPH), n (%)**			**6 (25%)**	
**iPCa-Gleason score 6, n (%)**			**6 (25%)**	
ISUP 1			6 (100%)	
**csPCa-MRI-visible, n (%)**			**6 (25%)**	
ISUP 2			3 (50%)	
ISUP 3			3 (50%)	
**csPCa-MRI-non-visible, n (%)**			**6 (25%)**	
ISUP 2			4 (66%)	
ISUP 3			2 (33%)	
**Renal function parameters (group-specific values)**				
**Variable**	**BPH,** **mean (IC95%)**	**iPCa Gleason 6, mean (IC95%)**	**csPCa-MRI-visible,** **mean (IC95%)**	**csPCa-MRI-non-visible,** **mean (IC95%)**
Creatinine (mg/dL)	0.93 (0.71–1.14) (median: 0.91)	0.88 (0.67–1.09) (median: 0.97)	0.98 (0.75–1.22) (median: 0.98)	1.02 (0.82–1.2) (median: 1)
eGFR (mL/min/1.73 m^2^)	90 (median: 89.5)	99.2 (median: 82.5)	76 (median: 81)	80.6 (median: 73)

**Table 2:** Functional annotation of the 82 selected proteins based on UniProt categories.

Functional Category	UniProt Codes
Cytoskeleton and motility	O75503, P59998, P07360, Q07075, P13797, P26447, P55083, P13796, P05787, P08729, Q9BXS5
Translation/RNA	P05387, P05386, P15586, O00264, P38571, Q12841, Q14108, P13639, P62330, Q9Y3B3, Q14240
Metabolism/Redox	Q02083, P23526, P22392, Q00796, P00338, P00441, P09417, P14618
Signaling and regulation	P22352, P06454, Q9BY67, Q14847, P08294, P08582, Q02952
Cell adhesion/ECM	Q12860, Q16610, P51884, P05556, P16070, P43121
Immunity/Inflammation	P80188, P05362, P04439, P08236, P06702, P19801
Vesicle trafficking/Endocytosis	Q9UMX5, Q7Z3B1, Q9BRA2, P51149, P08962, P11234
ER stress/Protein folding	O43598, Q14894, P34932, Q9UM22, P18827
Protease regulation/Innate defense	P30740, P07384, P21291, P81605
Lipid/Small-molecule transport	P02753, P05413, P02654, P12724
Ubiquitin-proteasome system	Q9BRT3, Q9Y5K6, O14618
Cell cycle/DNA replication and repair	P41222, Q9NR45
Cell cycle/Nucleotide metabolism	P61916, P07996
Mitochondria/Stress response	Q15185, Q92485
Peptidase/Extracellular processing	P12821, P08473
Lysosome/Autophagy	P13473
Membrane organization	Q9UQB8
Cell death/Differentiation	P31944

## Data Availability

The proteomics data generated in this study have been deposited in the PRIDE repository under accession number PXD074635.

## Supplementary Materials

The following supporting information can be downloaded at: https://www.mdpi.com/article/10.3390/life16030383/s1, Figure S1: Tissue and urine log2-transformed intensity correlation analyses for LCN2 (P80188), S100A9 (P06702), and AOC1 (P19801); n = 12 paired samples. A scatter plot displays each patient’s value and its regression line. Pearson correlation coefficients and *p*-values are shown. The analysis reveals modest agreement for LCN2 and matrix-dependent variability for AOC1; Table S1: Differentially expressed proteins identified in urine and tissue samples (Excel file), including protein accession numbers, gene names, log2 fold changes, and adjusted *p*-values (FDR correction).
