# Supplementary figures and images for: Integrated Urinary and Tissue Proteomic Signatures Reveal Core and Progression Biomarkers in MRI-Visible and MRI-Non-Visible Prostate Cancer
Source: Life (Basel). 2026 Feb 27;16(3):383. doi: 10.3390/life16030383 (PMC13028000; doi:10.3390/life16030383)

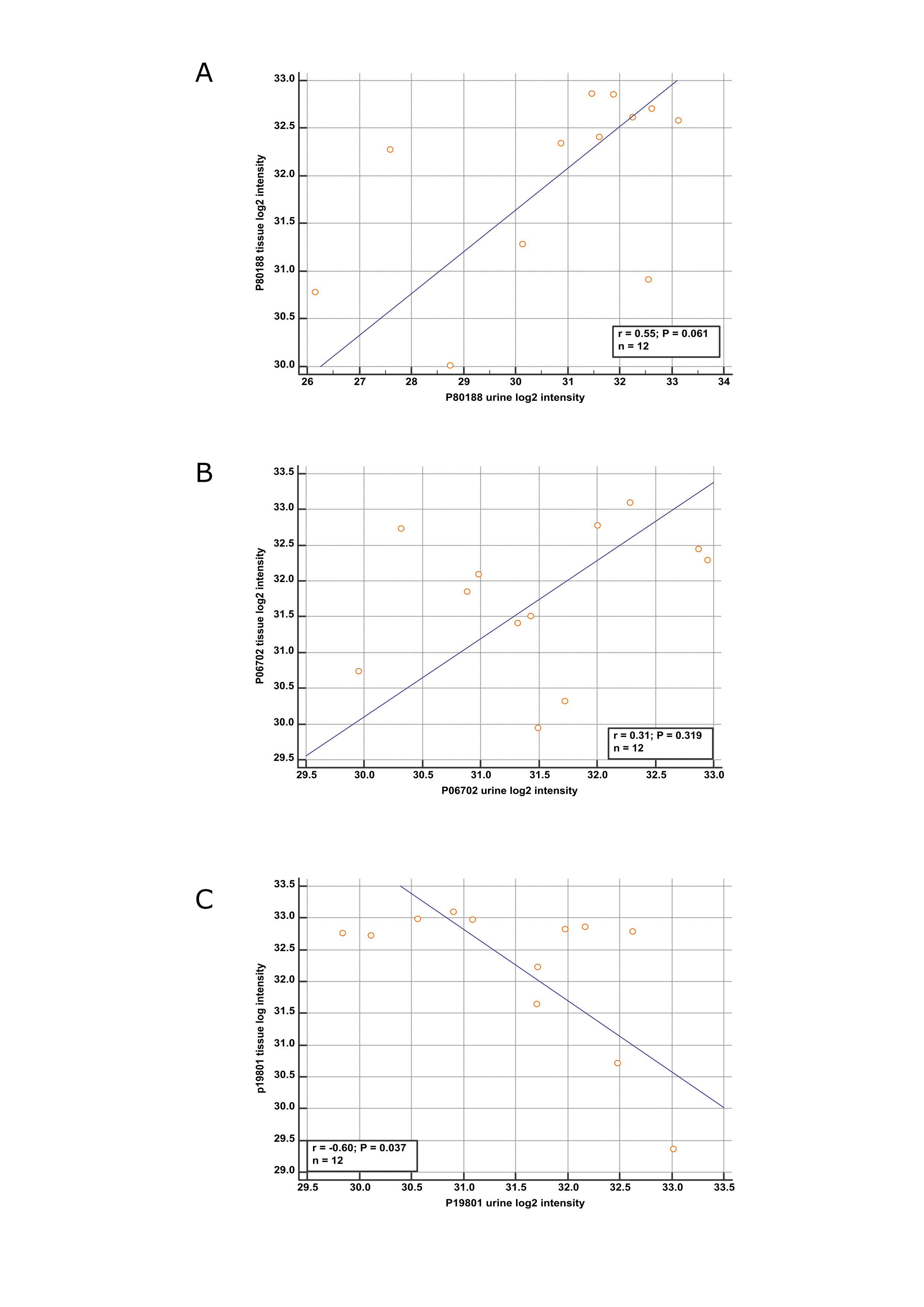

Supplement: Supplementary file 1 [file life-16-00383-s001.zip › Supplementary Figure S1.tif]
